# Dopamine and Octopamine Influence Avoidance Learning of Honey Bees in a Place Preference Assay

**DOI:** 10.1371/journal.pone.0025371

**Published:** 2011-09-30

**Authors:** Maitreyi Agarwal, Manuel Giannoni Guzmán, Carla Morales-Matos, Rafael Alejandro Del Valle Díaz, Charles I. Abramson, Tugrul Giray

**Affiliations:** 1 Department of Chemistry, University of Puerto Rico, San Juan, Puerto Rico; 2 Department of Biology, University of Puerto Rico, San Juan, Puerto Rico; 3 Laboratory of Behavioral Biology and Comparative Psychology, Oklahoma State University, Stillwater, Oklahoma, United States of America; AgroParisTech, France

## Abstract

Biogenic amines are widely characterized in pathways evaluating reward and punishment, resulting in appropriate aversive or appetitive responses of vertebrates and invertebrates. We utilized the honey bee model and a newly developed spatial avoidance conditioning assay to probe effects of biogenic amines octopamine (OA) and dopamine (DA) on avoidance learning. In this new protocol non-harnessed bees associate a spatial color cue with mild electric shock punishment. After a number of experiences with color and shock the bees no longer enter the compartment associated with punishment. Intrinsic aspects of avoidance conditioning are associated with natural behavior of bees such as punishment (lack of food, explosive pollination mechanisms, danger of predation, heat, etc.) and their association to floral traits or other spatial cues during foraging. The results show that DA reduces the punishment received whereas octopamine OA increases the punishment received. These effects are dose-dependent and specific to the acquisition phase of training. The effects during acquisition are specific as shown in experiments using the antagonists Pimozide and Mianserin for DA and OA receptors, respectively. This study demonstrates the integrative role of biogenic amines in aversive learning in the honey bee as modeled in a novel non-appetitive avoidance learning assay.

## Introduction

Throughout the past century the honey bee has been used as a model organism for neuroethological and behavioral studies including behavioral physiology, communication, navigation, social organization, learning, and memory [1]–[3]. For the past 50 years the focus of learning studies in honey bees has been the PER, or proboscis extension response where positive or negative appetitive associations that harnessed bees show were studied (see [4]). Recently, with further understanding of learning and memory mechanisms and developments in neurobiological and genomic understanding of the bee model, there is interest in developing novel laboratory assays and assays for individuals that are not harnessed to test mechanistically other modalities in honey bee learning and memory [2], [5]–[8]. We combined concepts from the Kolmes [9] sting response (SR) assay with the Vergoz et al. [2] SER or sting extension reflex conditioning assay to test avoidance conditioning of individual walking honey bees. We used an aversive stimulus that does not elicit sting extension (6 V, 50 mA electric current applied to half of a Kolmes electric grid). This way we were able to test multiple individuals without interference due to alarm pheromone and venom release that would accompany stimuli leading to sting extension. Bees in individual lanes walked across a grid and associated a colored location with electric shock punishment.

Avoidance conditioning in invertebrates has been studied with two paradigms [10], [11]. In the signaled avoidance paradigm, invertebrates are trained to avoid or postpone the aversive event by responding to a cue. In the punishment paradigm (also known as passive avoidance, or place avoidance) the animal avoids the aversive event by not entering a location, or emitting a response, that produces the aversive event.

Signaled avoidance has been demonstrated in free flying honey bees [12], green crabs [13], and earthworms [14]. It has been less convincingly demonstrated because of a lack of control procedures in cockroaches [15], planarians [16], and crayfish [17]. Punishment or passive avoidance has been investigated in a number of insects including cockroaches in a yoked-design for leg shock [18], [19], ants terminating substrate vibration [20], honey bees confined to a shuttle box with exposure to formic acid as the aversive stimulus [12] or with harnessed bees using the PER paradigm [21]. The Smith et al [21] experiment had the interesting property that bees received two conditioned stimuli both paired with sucrose but responding to one was punished with shock. Bees learned to extend their proboscis to the conditioned stimulus followed by food but to withhold proboscis extension to the conditioned stimulus paired with food and if it responded, shock.

In nature, bees and other organisms face a combination of rewards and punishers and respond appropriately by approaching or escaping these stimuli. Although previous studies based on pharmacological methods suggest that DA is involved in punishment learning and OA in reward learning in honey bees and other insects, recent fine scale neural studies show that this is a simplification and these biogenic amines are not necessarily specific to one pathway, probably reflecting an evolutionary common past starting with motor neuromodulation (rev. in [22] and see below). Insects, especially honey bees with a rich repertoire of vertebrate-like behavior, have provided an excellent experimental model that is mechanistically and conceptually relevant for molecular cognition research [23]. This is the first study where the new avoidance paradigm is used to determine possible effects of both DA and OA on aversive learning in honey bees.

At the molecular level, the mechanisms of learning are shared between vertebrates and invertebrates. In mammals, midbrain dopaminergic neurons participate in reward learning of a wide range of visual, auditory and somatosensory stimuli and thus are considered to serve as a general reward system [24]. It is likely that the roles of aminergic reinforcement systems in associative learning of a variety of sensory stimuli are conserved across different phyla, although there is also a notable difference that dopamine principally mediates appetitive reinforcement in other taxa but it mediates principally aversive reinforcement in the insects *Apis mellifera* [2], *Gryllus bimaculatus* [25], and in many instances in *Drosophila melanogaster* ([26], but see [27], [28]).

In the central nervous system (CNS) of both vertebrates and invertebrates, biogenic amines control and regulate various vital functions including circadian rhythms, endocrine secretion, circulatory control, as well as learning and memory. In insects, amines like DA, tyramine (TYR), OA, serotonin, and histamine exert their effects by binding to specific membrane proteins that primarily belong to the super family of G protein-coupled receptors. For example, the role of OA in insects serves a similar function to the general arousing role of adrenaline in vertebrates [29]. Moreover, OA is known to be involved in motivation, reward, and modulation of motor functions in insects [26]. There also is a group of related chemicals that are useful in the pharmacological study of this biogenic amine. Previous studies have shown that mianserin acts as an antagonist of OA receptors and thus blocks the effect of OA in the honey bee brain [30], [31]. Although mianserin is also known to block serotonin receptors, it is a potent insect neural octopamine receptor antagonist [32] used in other behavioral studies on honey bees as OA antagonist (e.g. [33]). We also have access to a direct precursor, tyramine, which is a functional neuromodulator [34]. As for DA, previous experiments with fruit flies carrying temperature-sensitive alleles of the dopa-decarboxylase gene involved in the biosynthesis of DA has indicated a role of this biogenic amine in electric shock olfactory learning [35].

Honey bees serve as an excellent model in understanding the functional role of octopaminergic and dopaminergic receptors and their intracellular signaling systems [36]. For example, receptors for DA (AmDOP1) [37], TYR (AmTYR) [38], and OA (AmOCT1) [39] are all present in the brain of honey bees. That these metabotropic receptors are involved in aversive learning and memory in bees has been tested by studying the effect of the octopaminergic and dopaminergic antagonists (to block their receptors) in the learning and memory tests performed using the SER paradigm [2]. However, in the study of Vergoz and colleagues [2], an interaction between reward pathway and aversive learning was not explicitly tested, instead their distinct nature was studied by conditioning either appetitive positive response (PER) or the aversive negative response (SER) in the same individual to distinct stimuli. These results are consistent with studies on other insects where biogenic amine effects on aversive and reward learning has been studied [25], [26]. However, there could be further integration across the distinct pathways should both DA and OA signals were present at the same time. The complex integration could be exemplified in a recent study where human subjects viewing the pictures of a romantic partner, which is linked to activation of the reward system, tolerated higher levels of thermal pain (e.g. [40]).

In this study we investigated the effects of both DA and OA on aversive learning in honey bees, both antagonist and agonists for DA and OA receptors were given to subjects: OA , DA , tyramine (precursor of OA), mianserin (to counter the effects of OA), and pimozide (to counter the effects of DA). We examined the effects of these treatments on the learning curves, punishment time or time spent in shock area, and the proportion of bees trained not to enter a compartment associated with the presentation of shock. A hypothesis on how punishment and reward pathways may interact during aversive learning is discussed.

## Results

### Avoidance behavior

**Figure 1:**
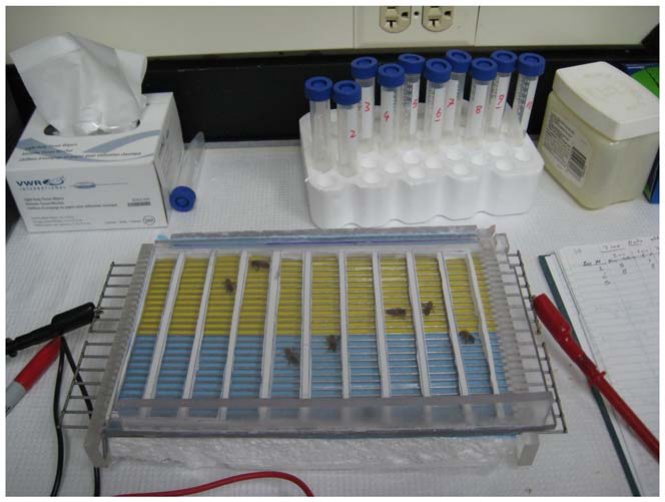
Place preference assay apparatus. Bees are placed in individual lanes, sandwiched between a Plexiglas® cover and a metal grid that is electrified (6 V, 50 mA) on one half. The halves have a color card placed under the grid. In this picture the yellow half is electrified and blue half is “safe”. Bees develop preference for the safe half, they show avoidance to the color side associated with electric shock. To prevent bees from walking upside-down and avoiding the shock, the Plexiglas® cover is coated with a thin layer of Vaseline, using a wipe. Bees are transported in 15 ml numbered culture vials and followed individually. Both manual records of position at every 15 secs and video recording and transcription are used for data collection.

The experiments were conducted in a new apparatus that resembles a shuttlebox (Figure 1). Honey bees learn to associate a mild electric shock with a spatial color cue. After a number of shock-cue associations, the honey bee restricts its activity to the side of the shuttlebox associated with the non-shock color. Details of the apparatus are provided in the Methods Section.

### Avoidance conditioning

**Figure 2:**
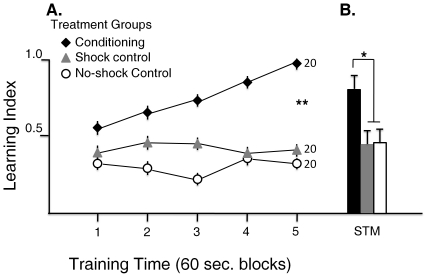
Learning and memory tests for bees in the groups for avoidance conditioning, none-shock control, and shock control. The avoidance conditioning treatment served as the conditioning control in other experiments where influence of biogenic amines on learning and memory were tested. **A. Learning:** Bees in the avoidance conditioning group demonstrate a significant learning effect as indicated by increase in learning index over the 5 minute training period. This is in contrast to the none-shock and shock control groups with no evidence of improved avoidance where either no shock was applied or shock was applied to both halves of the apparatus. In repeated measures MANOVA, the conditioning group vs the control groups are significantly different (F = 11.09, df = 2, P<0.001). The change in learning index over time showed significant difference based on group (Repated measures MANOVA, time blocks (5) and groups (3) interaction: F = 2.28, df = 8, P<0.03). **B. Short term memory (STM) test**: In the STM test performed 20 minutes after the second training bout, the conditioning group bees scored high for learning index (mean ± SE = 0.80±0.09, N = 20) similar to the end of training period, and statistically significantly different than the control group bees ( none-shock control: mean ± SE = 0.46±0.1, N = 20; shock control mean ± SE = 0.44±0.1, N = 20; ANOVA for STM learning index: df = 2, F = 3.9, p<0.03).

Time spent by bees on either half of the apparatus was not influenced by color in the absence of electric shock (none-shock control) or when electric shock was uniformly applied to both sides of the grid (shock control). When shock was presented on only one side of the apparatus, bees learned over time not to enter the section with the color cue (either blue or yellow in different experiments). The learning curves were plotted based on a learning index or time the bee stays in none-shock section in relation to total time interval or block, calculated as described in the Methods (Figure 2, in 60 sec. blocks). This learning index reports the differential preference for the none-shock area. In case of no preference for either area or no learning the individual would spend equal time on each side and would score 0 for that interval. The individual can maximally score 1. Reduction in time spent in the shock area is not considered as “learning” until a clear preference for the none-shock area becomes evident. Repeated measures ANOVA confirmed that bees would decrease entering the blue or the yellow section of the grid if that section was associated with shock and that performance improved over the course of the 5 minute training session relative to paired controls. The paired controls did not show a change over the 5 minute period for time spent on either color section (Figure 2a, see legend for statistics).

After a 10 minute intertraining interval, a second 5 minute training was performed where results were similar for bees in shock and none-shock control groups, and conditioned bees remained at or near maximal learning index through the training (results not shown). After a 20 minute wait period bees in all groups were tested for place preference (see Methods). The conditioned bees preferred the color not associated with shock significantly more than the controls, indicating presence of a short term memory (STM; Figure 2b).

### Biogenic amines and avoidance conditioning

To test the central hypothesis that reward and punishment pathways interact in aversive learning we examined the effect of the biogenic amines DA and OA and their respective antagonists (mianserin for OA, and pimozide for DA) on avoidance conditioning. We chose pimozide as the DA antagonist because this is a specific vertebrate D2 type receptor blocker, and in previous studies the best results on blocking aversive learning had been obtained by either broadly active antagonists such as Flupentixol or D2 antagonists such as Spiperone (see [2]). One caveat is that there is currently no information available about the specificity of pimozide action in insects. The chemical treatments were dissolved in sucrose syrup and made continuously available to bees for approximately 12 hours (overnight). The feeding method is easy and allows treating multiple individuals simultaneously, without anesthesia; and later allows animals to be tested even when not harnessed, in different laboratory and field behavioral assays [41], [42]. This method is known to result in an increase in brain biogenic amine levels in insects [41], [43], and in many experiments, long-term feeding resulted in behavioral effects even when entire colonies were treated with biogenic amines [33], [36], [41]–[43].

We used a 1 mg/ml dose for all neurochemicals. This dose has been shown to result in behavioral effects for TYR, OA, mianserin, and DA antagonists [36], [41]. We also examined the responses to lower and higher doses to determine any specific dose effects to the neural activity of the compounds (see Methods).

The DA treatment experiments tested the known association of DA, thought to be involved in punishment pathway found in the SER paradigm (see [2]) and determined if the effects were similar in the new place preference-based avoidance learning task. Because effects of DA on learning performance were positive and confirmed previous results, and locomotion during the assay were not different across the DA and control groups (results not shown), we did not find it necessary to test potential differences in walking activity of bees [44] that were treated with DA or antagonist in the learning assay box. The OA experiments examined the interaction of reward pathway with punishment learning using the same avoidance paradigm. Because octopamine is known to alter motor and sensory responses in other conditions [45], [46], we performed experiments to test for peripheral effects of OA under current assay conditions. These experiments showed that peripheral activity did not confound effects on aversive learning because OA treated bees and sucrose-fed bees in the control group had similar locomotor activity in the assay chamber lanes, and they showed sting reflexes at statistically similar stimulus (V) levels (see Figure S1 a , b).

We report the results of four experiments where bees in treatment groups were compared for: 1. Learning Curve: Improvement in avoidance over time 2. Punishment time during avoidance training 3. Dose responses and dose specific effects of biogenic amines 4: Proportion of forager bees trained to complete avoidance in learning tests.

#### 1. Learning Curve: Improvement over time for avoidance learning

**Figure 3:**
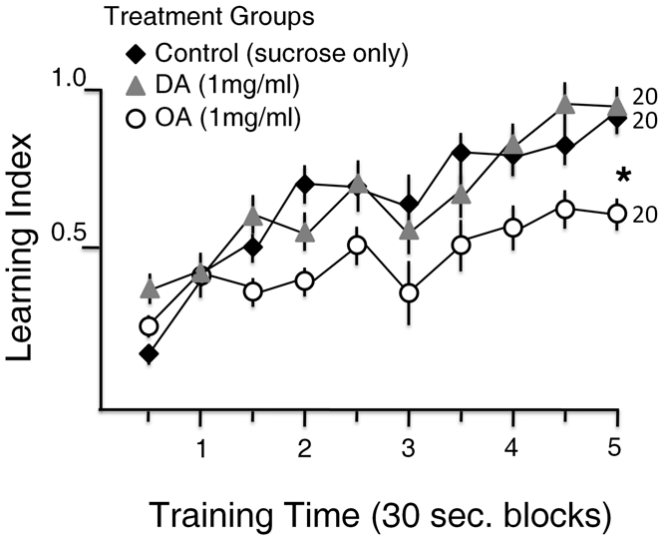
Avoidance conditioning learning curves. Bees in the principal treatment groups (control, OA, and DA) all showed increase in learning index over time (Repeated measures MANOVA, time −10 blocks-: F = 13. 25; df = 9; P<0.001). OA group bees showed a significantly slower increase and reached a lower maximum learning index (Repeated measures MANOVA, groups (3): F = 4.06, df = 2, P<0.025).

In an experiment we compared the time course of learning for the three main treatments: sucrose-fed bees (the treatment control), DA treatment and OA treatment bees. A learning curve was plotted for each group of bees showing the learning index (over 30 sec blocks) for individuals over the first 5 minute training time. The learning index is defined above, and calculated as described in the Methods. The learning curves were compared across groups. Bees treated with DA and control bees displayed similar learning curves in the first 5 minute training (Figure 3). It was found that the bees treated with OA learned slower compared to bees in the DA-treatment or control groups (Figure 3). In the second 5 minute training the learning indices for different groups did not change, remained similar to end of first training learning indices for each group (at minute 5 of second training, mean ±SE for control: 0.93±0.04; for DA: 0.99±0.06; for OA: 0.78±0.08; ANOVA df = 43, F = 2.5117; P = 0.10).

#### 2. Punishment time during avoidance training

**Figure 4:**
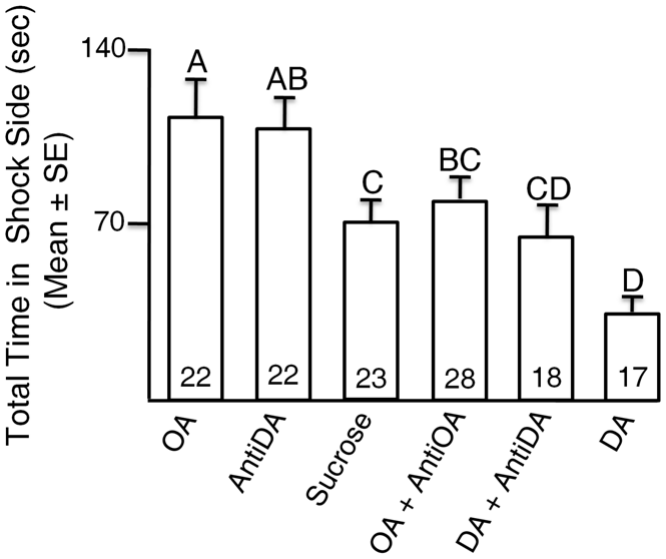
Punishment time during learning. The total time bees were on the shock half of the apparatus during the 5 minute training session is the punishment or “total time in shock side” during learning. Punishment time during learning for groups of bees fed sucrose (Control), or sucrose with treatment chemicals (OA, DA, OA+ Mianserin, Pimozide, DA+Pimozide) are shown. There are statistically significant effects of treatment on punishment time during learning (Factorial ANOVA for treatment group effect: F = 6.4083, df = 5, P<0.0001). The numbers in the bars indicate the number of individuals in each group. The bars with different letters are statistically significantly different (P<0.05) from each other in multiple comparisons.

Time spent in the shock area during avoidance training in the first training period were compared for bees treated with biogenic amines and the sucrose-fed bees in the control group in a factorial ANOVA. Time spent in the shock area during the training period was greater for bees treated with OA in comparison to bees in the control group. Time spent in the shock side was less for bees treated with DA in comparison to sucrose-fed bees. These differences were statistically significant (p<0.05, Tukey's multiple comparison tests; Figure 4). Pimozide treated bees also spent more time in the shock section. Mianserin reversed the behavioral phenotype of OA treated bees and made their performance similar to control bees with regards to the time spent on the punished side during the first 5 minute training session. Administering both DA and pimozide compounds countered each other's effects, resulting in punishment times similar to the sucrose-fed bees.

#### 3. Dose responses to biogenic amines

**Figure 5:**
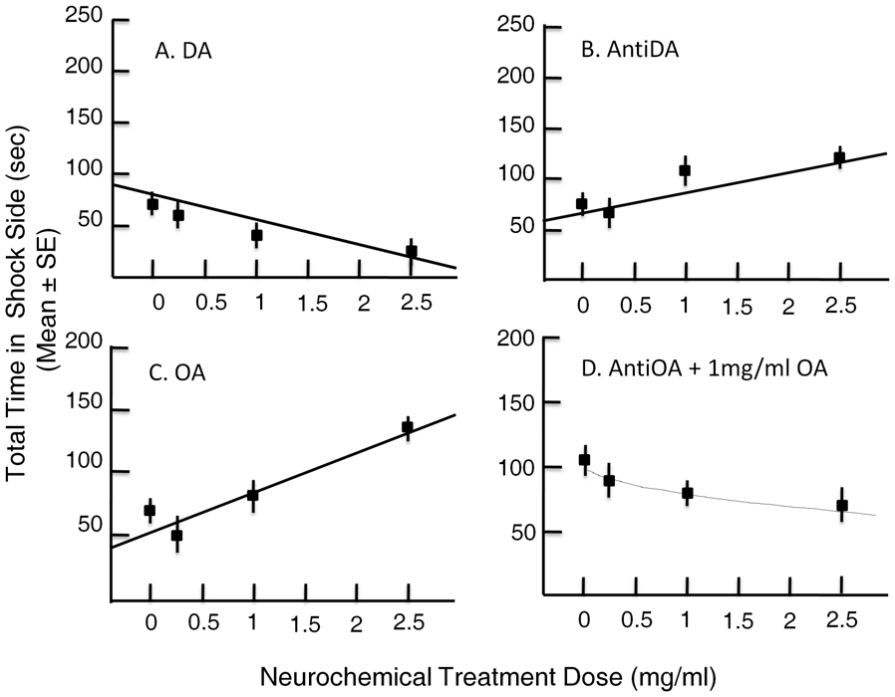
Dose responses for total punishment (shock) time during avoidance conditioning. **A. DA dose response.** A linear fit (y = 78.152−23.226x; r = 0.45; n = 35, P<0.01 where y = punishment time in sec. and x = dose in mg/ml) shows increasing dose of DA to lead to reduced punishment time during avoidance conditioning. **B. Pimozide dose response.** A linear fit (y = 67.784+19.606x; r = 0.39; n = 43, P = 0.01) shows increasing dose of Pimozide to lead to increased punishment time during avoidance conditioning. **C. OA dose response.** A linear fit (y = 57.472+30.169x; r = 0.52, n = 40, P<0.01) shows increasing dose of OA to lead to increased punishment time during avoidance conditioning. **D. Mianserin dose response.** In the case of mianserin, only the square root fit (y = 102.167−23.172x^1/2^; r = 0.31, n = 50, P<0.05) was statistically significant, indicating a potential titration of OA effect beyond the 1 mg/ml mianserin dose.

Experiments were performed using four doses of biogenic amines and their antagonists (0, 0.25, 1, and 2.5 mg/ml doses) in 2 M sucrose solution. For DA, pimozide, and OA the 0 dose group were only fed 2 M sucrose. To test dose response to mianserin we fed bees with 1 mg/ml OA in 2 M sucrose solution, and simultaneously included in the same solution 0, 0.25, 1, or 2.5 mg/ml mianserin . The rationale for this approach was based upon the work of Vergoz et al. [2] who showed that mianserin alone did not influence aversive learning, and our pilot studies confirmed this observation (data not shown). Regression analyses were done for the time spent on the punished side during training for different treatment doses. A linear curve fit, and a square root transformed fit (indicative of saturation) for regression of time spend in punishment side to dose were explored for statistical significance (Figure 5 A, B, C, D). Except for mianserin, all treatments demonstrated a statistically significant linear fit not significantly different from a transformed fit, with statistical power ≥0.9. Higher DA treatment doses led to lower time spend in the punishment side during training; the slope was negative (Figure 5A). Higher doses of pimozide led to longer punishment times during training, the slope was positive (Figure 5B). Higher OA treatment doses also led to longer punishment times during training, the slope was positive (Figure 5C). In the case of mianserin, only the square root fit was statistically significant, indicating a potential titration of OA effect beyond the 1 mg/ml mianserin dose. This result is consistent with the mianserin blocking effect of OA in this avoidance assay (Figure 5D).

#### 4. Proportion of forager bees trained to complete avoidance of shock side

Bees that learned not to enter the shock area, and have not made mistakes (entered the shock section) in the last 90 seconds of second training were considered to be “trained to complete avoidance”. Although all bees show improvement over time, not all were trained to this strict learning criterion at the end of second training period. Lastly, we also examined proportion of genetically similar vs. dissimilar honey bee foragers trained to complete avoidance in the place preference assay, as a means to test influence of genetic variation on individual variation in learning performance (Figure S2).

**Figure 6:**
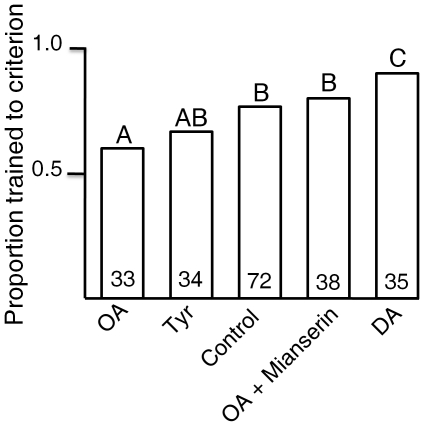
Training to criterion of complete avoidance. Proportion of bees trained to complete avoidance across treatment groups (control, OA, DA, TYR, and OA+ Mianserin) differed significantly (Likelihood ratio test: X^2^ = 13.458, df = 4, P<0.01). The numbers in the bars indicate the number of individuals in each group. The bars with different letters are statistically significantly different (P<0.05) from each other in multiple comparisons.

We subjected comparable groups of bees to avoidance conditioning (35 to 50 individuals per colony per treatment). A smaller proportion of the bees treated with OA were successfully trained to complete avoidance in comparison to control bees. A greater proportion of bees treated with DA were successfully trained in comparison to the bees in the control group. These differences were statistically significant (p<0.05; Figure 6A). The performance of tyramine treated bees was between the OA and sucrose control, not significantly different from either. The effect of OA was specific, as it was reversed by the mianserin. In this particular test we did not examine the effect of pimozide.

## Discussion

The most significant conclusion of this study is that biogenic amines thought to be associated either with reward or punishment pathways of honey bees, both influence learning in a non-appetitive avoidance situation. The new place preference assay provides a method for rapidly conditioning multiple individuals simultaneously to avoid an area with a color cue associated with electric shock punishment. This assay adds to the arsenal of different conditioning protocols to access the neurobiology of learning in the honey bee model (e.g. PER, SER, artificial flower visits). Treatment with the biogenic amine shown to be necessary for reward learning (see [8]), OA, had a negative effect on punishment learning, in that a lower proportion of subjects reached complete avoidance and they spent more time on the punished side than bees either receiving treatment with DA, or bees in the control group, not receiving any external biogenic amines.

This honey bee shock avoidance assay is reminiscent of the olfactory shock avoidance assay for *Drosophila* [47], however there are certain differences. First, in this assay individual bees, rather than populations of individuals, are used permitting greater control of training variables. Second, in the *Drosophila* assay olfactory shock avoidance was tested in a T-maze following the odor-shock treatment. In the bee procedure, we measure avoidance conditioning directly. Third, in the *Drosophila* assay, the unconditioned response to light is used to get the flies to move onto the shock grid. In the bee assay no such procedure is used. In fact, inspired by this bee avoidance assay, Dr. Jose L. Agosto is currently developing an individual-based conditioned place preference assay for flies (personal communication).

The effects of both DA and OA on punishment learning leads to a hypothesis that interaction between reward and punishment pathways could be important in decision making. For instance, a bee needs to learn to avoid entering the wrong colony, weigh the cost of visiting a nectar source against the lack of receiver bees at the colony [48], or decide to communicate a patch where predators were encountered [49] or visit reward sources that may not be constant [50], [51]. In light of current results we do see the need to test the hypothesis also suggested by Vergoz et al. [2] that neuromodulation by aminergic neurons serves as a value system in associative learning, i.e. as a system for valence and salience assignment to stimuli to be learned [23], [52]. We suggest that one possibility for integrating negative and positive values of a complex situation could be accomplished at the memory acquisition phase. Rewarding stimuli associated with the situation may decrease the salience of negative stimuli [52]–[54], and subsequently influence learning of the association. For instance, an unreliable reward from a particular flower may lead to further visits to flowers of this color even in the absence of reward (see [51]). Another example of integration of punishment and discriminative reward learning is exemplified with the taste and taste aversion study where free flying bees were able to better recall reward associated color in contrast to a repellent associated distractor [8].

In fact, our first expectation based on other studies in honey bees was that OA would have no effect on punishment learning in the honey bee. Therefore, when we observed the negative effect, we explored and tested alternate explanations. The experiments performed to test whether the effect of the biogenic amine OA on learning performance were confounded by peripheral effects demonstrated that bees not treated with biogenic amine (i.e. the sucrose-fed bees in the control group) showed a similar sensitivity to the electric shock and similar locomotor activity to that of the bees treated with OA. We conclude from these findings that the effect of OA on aversive learning is not in the peripheral nervous system, or perception and locomotion but is in the central nervous system. This influence of OA on aversive conditioning is compatible with documented roles of OA on defensive behaviors in honey bees [55].

We also tested the possible effect of the precursor for OA and found that TYR treated bees to be less likely to avoid the color associated with shock treatment compared to sucrose-fed control bees; although this difference was not statistically significant. Recently published studies demonstrated the presence of a specific tyramine receptor in the honey bee brain ([38], see also [36], [56]) and this complicates any allocation of a tyramine effect to OA, but does open the possibility of several pathways interacting in an integrative fashion.

Overall results show that exposure to OA results in lower punishment learning performance and DA in better punishment learning performance, and that these effects are specific to the biogenic amines. However, there were large differences in learning characteristics of individual bees, as noted in the proportion of bees that were trained to complete avoidance criterion. These differences could be due to size, past experience, age, genetic differences, pre-test feeding behavior, and in case of bees in the treatment group, the delivery of neuroactive chemicals to target tissues [57]–[60]. We found foragers to range between 75 to 115 mg, and in a test of sucrose-fed bees no significant correlation was found between weight and learning score of foragers (unpublished results, Arian Avalos, Tugrul Giray, Ivette Hernandez). We controlled for age and behavior of the test bees by using only confirmed forager bees. We controlled motivation and feeding by keeping bees in the incubator cages for 4 hours without feeding, and later feeding them *ad libitum* with 2 M sucrose solution (with appropriate treatment chemical) for approximately 12 hours until tested.

We also found that learning in genetically similar bees (g = 0.75) derived from a queen mother inseminated instrumentally with semen from a single male were not different from genetically dissimilar bees (0≥g<0.25) (Figure S2; see [61], [62]). We conclude from these results that variations in the behavior of bees fed different biogenic amines during aversive learning do not depend upon the behavior, age, and genetic differences. The use of a delivery method that is more precise and targeted, or altering target receptors by molecular methods such as RNAi [44], [63]–[65], could lead to more uniform results. Given these variations, the results were robust for the effect of DA, OA and their antagonists on avoidance conditioning. These results also attest to the robust and reliable nature of the new place preference assay. The assay has already been adopted for effects of ethanol on learning in honey bees (Charles Abramson, unpublished results), molecular correlates of learning and long term memory in bees (Sandra Peña de Ortiz, unpublished results) and for potential learning differences in diseased and healthy bees (Elizabeth Capaldi, personal communication).

One insight from this research is the importance of using different learning protocols to access experimentally the neuropharmacology of learning. In addition, the learning assay that we have used in these experiments can be adopted as a test system for the evaluation of candidate drugs and potential hazardous chemicals for humans or other animals [53], [66]. More fundamentally, this research resulted in a novel hypothesis on an integrative understanding of reward and punishment pathways in aversive learning.

## Materials and Methods

### The bees

Honey bees were maintained in typical colonies at the Gurabo Agricultural Experimental Station of the University of Puerto Rico according to standard beekeeping practices. Bees for the current experiments were captured from several colonies to have a representative genetic sample of individuals. We specifically collect foragers that are typically older bees (21–30 days of age) with experience in foraging tasks that require learning locations, colonies and navigation in the field (e.g. [23]). Once captured, the bees are placed in a holding cage for four hours, treated overnight in individual feeding cages (JZBZ™ queen cages) and later tested for learning and memory within one day of removal from the source colony. When in the laboratory, bees are maintained in an incubator simulating colony conditions: dark, 34±1 degrees C, and >80% relative humidity.

### Treatment method

We orally administered different biogenic amines and their corresponding antagonists. This method of drug application was preferred over other methods as it does not require that the animal be anaesthetized or harnessed as is the case if the drug is given by injection (e.g. [2]). Also a large number of bees can chronically receive drug administration in contrast to other methods [60].

In the present experiment, the appropriate amount of drug was dissolved in 2 M sucrose solution. Bees, on average, consumed 40 µl of this solution overnight (see [41]). The next day, for each assay ten bees at a time were randomly chosen for training and tests.

### The apparatus

Honey bees were confined between a Plexiglas lid and a metallic grid [9]. The lid was coated by a thin layer of petrolatum jelly to prevent bees from walking upside-down. This allows bees to always be in contact with the grid. The grid is formed by two electrodes with wire extensions (2 mm in diameter). The space between the wire extensions is .35 cm. The electrodes were cut in the midpoint to form two halves that could be independently electrified. Just below the grid was a colored surface. To prevent the bees from somehow marking the colored surface a plastic wrap was placed beneath the wire extensions, and replaced after each training period. In this study we used only blue and yellow color cues, since these are reliably distinguished by honey bees [67]. In order to run several bees simultaneously, the Kolmes grid was further modified by creating 10 individual lanes made from poster board that were also replaced after one use. The lanes were 15 cm long by 2 cm wide. When placed within a lane an individual honey bee would repeatedly walk end to end most likely searching for an exit.

The electric shock was presented to only one side of the apparatus identified by a specific color. The shock was 6 V, 50 mA DC from an analog power source (see Figure 1). Bees in the electrified section of the apparatus quickly left this area. However, the voltage was low enough not to cause a sting reflex. The rationale for providing a mild shock was to prevent any interaction among bees in different lanes of the apparatus due to alarm pheromone potentially released with a sting extension reflex.

### Learning and memory test

Bees confined to assay tubes are anesthetized by placing the tubes on ice or by exposure to CO_2_. Anesthetized bees are placed in the center of the apparatus at the junction between the two colors and the apparatus moved to a dark incubator at 34 degrees C for about 10 minutes. When all bees are active, the apparatus is removed from the incubator and connected to a voltage source set at 6 V 50 mA, that delivers current to only one side with one of the two colors. Colors are counterbalanced in successive trials- half of bees learn to avoid yellow to avoid punishment, and the other half the blue.

In the present work, we used a visual cue in a avoidance conditioning paradigm where bees associate a color cue with a location where electric shock is administered upon entering. After a number of shocks the bee no longer enters the location. Whether this situation represents classical or instrumental conditioning and/or some combination cannot be said at this time. However, our procedure clearly has an instrumental component because the bee must make a response to receive the shock. The learned response is a decrease in entering a compartment associated with shock and shock is the aversive stimulus.

During the development of the avoidance assay, color pairs known to be distinct for honey bees (e.g. pink and white) were used as were colors known to be less distinct for bees (e.g. orange and green). Unpublished results indicate that bees train equally well to a wide variety of colors when they were associated with shock. Our rationale for using yellow and blue was because it is known that these colors are unequivocal signals for bees ([68]; rev. [67]).

### The assay consists of the following sessions

#### Two training sessions

Each session is 5 minutes long in which electric shock is given to either the yellow or blue section of the apparatus. The position of each bee is manually recorded every 30 seconds and time spent on each section transcribed from video recordings. After a session is completed bees are placed in an incubator for 10 minutes. The intersession period of 10 minutes was chosen to be twice the training period. These two training sessions separated by a 10 minute intersession interval correspond to the acquisition phase.

#### One test session/Short-term memory test

When required, short-term memory test is made 20 minutes after the last training session. Each session is of one minute duration. In this session, no electric shock is presented to the subjects. At the beginning of the test bees are placed on the side with the color not associated with shock. The position of each bee is recorded every 15 seconds and bees are videotaped. This test is related to the memory and recall phase and the short, 20 minute intersession interval, represents short term memory. In later phases of memory formation other long term processes as post-translational protein modifications such as phosphorylation and gene and protein expression may be important [6]. Our focus has been on the acquisition phase,most likely to be influenced by the biogenic amines.

### Learning Measurement

We used three measures of learning:

#### 1. Proportion of bees trained to criterion

This measures the total number of bees that stop entering the area associated with shock during the last 90 seconds of the second training session. Bees were considered “trained” if during the second training session bees stopped entering the compartment associated with shock. This varied across individuals, although most bees were successfully trained within the first 3 minutes of the first 5 minute training session. We decided to use “no mistakes in the last 90 seconds of second training” as a criterion in order to rapidly determine trained from untrained bees. This criterion also has the virtue of allowing a quick and conservative comparison of treatment effects on learning.

#### 2. Learning index

Based on the ratio of time at the none-shock side in each time block, a learning curve is constructed showing the change in learning index with respect to training time. For this index any value lower than 0.5 was considered 0, and index values between 0.5 to 1 were adjusted to a scale of 0 to 1, similar to a difference-based index for conditioned discrimination tests (see [2]). This method was used because control bees spend about equal time on the two sides of the apparatus in the absence of shock. Analyzing data in 30 second (for more detailed visualization of neurochemical effects) or 60 second blocks allows the use of parametric statistical tests such as repeated measures ANOVA [69]. A significant time or trial effect is a standard method to demonstrate presence of learning (e.g. [2], [70]).

#### 3. Punishment time during learning

A measure of the total time spent in the shock side during a training session. This measure of time spent at different parts of a place preference assay provides a simplified continuous measure of learning performance that can be compared across experiments (e.g. [71]). This parametric measure summarizes the learning performance of each individual over the 5 minute training period, and allows for the use of a factorial ANOVA to test for multiple treatment effects.

### Dose response curve

To determine the dose dependence of the effects of biogenic amines and interacting pharmacological agents, dose response curves were prepared for each drug. In this experiment we tested 0, 0.25, 0.5, 1 and 2.5 mg/ml doses to establish a dose response curve with respect to the time to learning measure (learning measure 3). This is because any negative effects on learning could be thought to be caused by toxic effects of the chemical treatment [72].

The dose response curve has helped us to analyze the involvement of specific brain receptors of the drug on the learning and memory processes in honey bees. We examined 20 bees per dose to examine the effect of different concentration of the drug on the behavior of bees. The sample size was based on power analyses and previous results (see, for instance, Figure 2).

### Statistical analyses

To compare treatment effects we used both the proportion of trained bees and the time to training. The data on the proportion of trained bees were compared using chi-squared tests. Data related to the time to training measure were compared in a factorial ANOVA. To measure learning and properties of learning curves, we used the learning index based on avoidance of color associated with shock. This allows us to perform repeated measures MANOVA, and improvement in performance of each bee could be followed over time. The statistical analyses were performed using the JMP™ statistical package from SAS.

## Supporting Information

Figure S1 **Locomotor activity and sting response threshold of bees.** **A.** Locomotor activity of bees in the OA treatment and control groups were statistically not different (t-test: t = −0.286; df = 28; P>0.77), measured as distance walked (cm) in unit time (30 sec.) by each bee in each lane of the assay chamber in absence of electric shock. **B.** Sting response threshold of bees in the OA and control treatment groups were statistically not different (t-test on log transformed data: t = 1.691; df = 28; P>0.10), measured as the least amount of electric shock (V) that resulted in sting extension response for bees tested individually in the assay chamber. The numbers in the bars indicate the number of individuals in each group.
(TIF)

Figure S2 **Genetic effects on variation in learning performance in the place preference assay.** Experiments were performed with genetically similar bees obtained from a queen that was instrumentally inseminated by semen from a single drone ( SDI colony, genetic relatedness coefficient g = 0.75) to see if the variations in learning were due to higher genetic variation found in typical colonies (typical colony, across colonies, g = 0). Comparison of SDI colony or typical colony bees for proportion of individuals trained to criterion in principal treatment groups (control, OA, DA), demonstrate that results are similar for both types of bees (Wald test: Genetic similarity: X^2^ = 0.174, df = 1, P = 0.68; Treatment: X^2^ = 18.969, df = 2, P<0.0001; Genetic similarity and treatment interaction X^2^ = 0.469, df = 2, P = 0.79). The main effects of treatments with OA where lower proportion of bees were trained to complete avoidance, and DA where higher proportion of bees were trained to complete avoidance, were similar in direction and magnitude to the pooled data for bees from different colonies with naturally mated queens.
(TIF)

## References

[1] Menzel R (2001). Searching for the memory trace in a mini-brain, the honey bee.. Learn Mem.

[2] Vergoz V, Rousel E, Sandoz JC, Giurfa M (2007). Aversive learning in honeybees revealed by the olfactory conditioning of the sting extension reflex.. PLoS ONE.

[3] Robinson GE, Fernald RD, Clayton D (2008). Genes and social behavior.. Science.

[4] Sandoz JC, Menzel R (2001). Side-specificity of olfactory learning in the honeybee: generalization between odors and sides.. Learn Mem.

[5] Carcaud J, Rousel E, Giurfa M, Sandoz JC (2009). “Odour aversion after olfactory conditioning of the sting extension reflex in honeybees.”. J Exp Biol.

[6] Giurfa M, Fabre E, Flaven-Pouchon J, Groll H, Oberwallner B (2009). Olfactory conditioning of the sting extension reflex in honey bees: memory dependence on trial number, interstimulus interval, intertrial interval, and protein synthesis.. Learn Mem.

[7] Menzel R (2009). Serial position learning in honey bees.. PLoS ONE.

[8] Avargues-Weber A, de Brito Sanchez MG, Giurfa M, Dyer AG (2010). Aversive reinforcement improves visual discrimination learning in free-flying honeybees.. PLoS ONE.

[9] Kolmes SA, Fergusson-Kolmes LA (1989). Stinging Behavior and Residual Value of Worker Honey Bees (*Apis mellifera*).. J New York Entom Soc.

[10] Campbell BA, Church RM (1969). Punishment and aversive behavior.

[11] Mackintosh NJ (1974). The psychology of animal learning.

[12] Abramson CI (1986). Aversive conditioning in honeybees (*Apis mellifera*).. J Comp Psyc.

[13] Abramson CI, Armstrong PM, Feinman RA, Feinman RD (1988). Signaled avoidance learning in the eye withdrawal reflex of the green crab.. J Exp Anal Behav.

[14] Abramson CI, Buckbee DA (1995). Pseudoconditioning in earthworms (*Lumbricus terrestris*): Support for nonassociative explanations of classical conditioning phenomena through an olfactory paradigm.. J Comp Psych.

[15] Chen WY, Aranda LC, Luco JV (1970). Learning and long- and short-term memory in cockroaches.. Anim Behav.

[16] Ragland RS, Ragland JB (1965). Planaria: Interspecific transfer of a conditionability factor through cannibalism.. Psychon Sci.

[17] Taylor RC (1971). Instrumental conditioning and avoidance behavior in the crayfish.. J Biol Psych.

[18] Disterhoft JF, Haggerty R, Corning WC (1971). An analysis of leg position learning in the cockroach yoked control.. Physiol Behav.

[19] Disterhoft JF (1972). Learning in the intact cockroach (*Periplaneta americana*) when placed in a punishment situation.. J Comp Physiol Psych.

[20] Abramson CI (1981). Passive avoidance in the California harvester ant.. J Gen Psychol.

[21] Smith BH, Abramson CI, Tobin TR (1991). Conditioned withholding of proboscis extension in honey bees (*Apis mellifera*) during discriminative punishment.. J Comp Psych.

[22] Barron AB, Søvik E, Cornish JL (2010). The roles of dopamine and related compounds in reward-seeking behavior across animal phyla.. Frontiers Behav Neurosci.

[23] Giurfa M (2007). Behavioral and neural analysis of associative learning in the honeybee: a taste from the magic well.. J Comp Physiol A.

[24] Wise RA (2004). Dopamine, learning and motivation.. Nat Rev Neurosci.

[25] Unoki S, Matsumoto Y, Mizunami M (2006). Roles of octopaminergic and dopaminergic neurons in mediating reward and punishment signals in insect visual learning.. Eur J Neurosci.

[26] Schwaerzel M, Monastirioti M, Scholz H, Friggi-Grelin F, Birman S (2003). Dopamine and octopamine differentiate between aversive and appetitive olfactory memories in *Drosophila*.. J Neurosci.

[27] Kim YC, Lee HG, Han KA (2007). D1 dopamine receptor (dDA1) is required in the mushroom body neurons for aversive and appetitive learning in *Drosophila*.. J Neurosci.

[28] Selcho M, Pauls D, Han KA, Stocker RF, Thum AS (2009). The role of dopamine in *Drosophila* larval classical olfactory conditioning.. PLoS ONE.

[29] Liberstat F, Pfluger HJ (2004). Monoamines and orchestration of behavior.. Biosci.

[30] Bischof LJ, Enan EE (2004). Cloning, expression and functional analysis of an octopamine receptor from *Periplaneta americana*.. Insect Biochem Mol Biol.

[31] Maqueira B, Chatwin H, Evans PD (2005). Identification and characterization of a novel family of *Drosophila* beta-adrenergic-like octopamine G-protein coupled receptors.. J Neurochem.

[32] Roeder T (1990). High-affinity antagonists of the locust neural octopamine receptor.. Eur J Pharmacol.

[33] Barron AB, Robinson GE (2005). Selective modulation of task performance by octopamine in honey bee (*Apis mellifera*) division of labour.. J Comp Phsyiol A.

[34] Nagaya Y, Kutsukake M, Chigusa SI, Komatsu A (2002). A trace amine, tyramine, functions as a neuromodulator in *Drosophila melanogaster*.”. Neurosci Lett.

[35] Tempel BL, Livingstone MS, Quinn WG (1984). Mutations in the dopa decarboxylase gene affect learning in *Drosophila*.. Proc Nat Acad Sci USA.

[36] Scheiner R, Pluckhahn S, Oney B, Blenau W, Erber J (2002). Behavioural pharmacology of octopamine, tyramine and dopamine in honey bees.. Behav Brain Res.

[37] Blenau W, Erber J, Baumann A (1998). Characterization of a dopamine D1 receptor from *Apis mellifera*: cloning, functional expression, pharmacology, and mRNA localization in the brain.. J Neurochem.

[38] Blenau W, Baumann A (2000). Amtyr1: characterization of a gene from honey bee (*Apis mellifera*) brain encoding a functional tyramine receptor.. J Neurochem.

[39] Grohmann L, Blenau W, Meadows B, Ebert PR, Baumann A (2000). Characterisation of an octopamine receptor from *Apis mellifera* (AmOCT1): cloning, functional expression and mRNA localisation in the brain.. Eur J Neurosci.

[40] Younger J, Aron A, Parke S, Chatterjee N, Mackey S (2010). Viewing pictures of a romantic partner reduces experimental pain: involvement of neural reward systems.. PLoS ONE.

[41] Giray T, Galindo-Cardona A, Oskay D (2007). Octopamine influences honey bee foraging preference.. J Insect Physiol.

[42] Barron AB, Maleszka R, Vander Meer RK, Robinson GE (2007). Octopamine modulates honey bee dance behavior.. Proc Nat Acad Sci USA.

[43] Schulz DJ, Sullivan JP, Robinson GE (2002). Juvenile hormone and octopamine in the regulation of division of labor in honey bee colonies.. Horm Behav.

[44] Mustard JA, Pham PM, Smith BH (2010). Modulation of motor behavior by dopamine and the D1-like dopamine receptor AmDOP2 in the honey bee.. J Insect Phys.

[45] O'Shea M, Evans PD (1979). Potentiation of neuromuscular transmission by an octopaminergic neurone in the locust.. J Exp Biol.

[46] Fussnecker BL, Smith BH, Mustard JA (2006). Octopamine and tyramine influence the behavioral profile of locomotor activity in the honey bee (*Apis mellifera*).. J Insect Physiol.

[47] Quinn WG, Harris WA, Benzer S (1974). Conditioned behavior in *Drosophila melanogaster*.. Proc Natl Acad Sci USA.

[48] De Marco RJ (2006). How bees tune their dancing according to their colony's nectar influx: re-examining the role of the food-receivers' ‘eagerness’.. J Exp Biol.

[49] Abbott KR, Dukas R (2009). Honeybees consider flower danger in their waggle dance.. Anim Behav.

[50] Seefeldt S, De Marco RJ (2008). The response of the honeybee dance to uncertain rewards.. J Exp Biol.

[51] Cakmak I, Song DS, Mixson TA, Serrano E, Clement ML (2010). Foraging response of Turkish honey bee subspecies to flower color choices and reward consistency.. J Insect Behav.

[52] Berridge KC, Robinson TE, Aldridge JW (2009). Dissecting components of reward: ‘liking’, ‘wanting’, and learning.. Curr OpinPharm.

[53] Berridge KC (2007). The debate over dopamine's role in reward: the case for incentive salience.. Psychopharmacol.

[54] Robinson TE, Berridge KC (2008). The incentive sensitization theory of addiction: some current issues.. Phil Trans R Soc B.

[55] Hunt GJ (2007). Flight and fight: a comparative view of the neurophysiology and genetics of honey bee defensive behavior.. J Insect Physiol.

[56] Lange AB (2009). Tyramine: from octopamine precursor to neuroactive chemical in insects.. Gen Comp Endocrinol.

[57] Arenas A, Farina WM (2008). Age and rearing environment interact in the retention of early olfactory memories in honey bees.. J Comp Physiol A.

[58] Harano K, Sasaki K, Nagao T, Sasaki M (2008). Influence of age and juvenile hormone on brain dopamine level in male honeybee (*Apis mellifera*): association with reproductive maturation.. J Insect Physiol.

[59] Page JRE, Erber J, Fondrk MK (1998). The effect of genotype on response thresholds to sucrose and foraging behavior of honey bees (*Apis mellifera* L.).. J Comp Physiol A.

[60] Barron AB, Maleszka J, Vander Meer RK, Robinson GE, Maleszka R (2007). Comparing injection, feeding and topical application methods for treatment of honey bees with octopamine.. J Insect Physiol.

[61] Giray T, Robinson GE (1994). Effects of intracolony variability in behavioral development on plasticity of division of labor in honey bee colonies.. Behav Ecol Sociobiol.

[62] Giray T, Robinson GE (1996). Common endocrine and genetic mechanisms of behavioral development in male and worker honey bees and the evolution of division of labor.. Proc Natl Acad Sci U S A.

[63] Farooqui T, Robinson K, Vaessin H, Smith BH (2003). Modulation of early olfactory processing by an octopaminergic reinforcement pathway in the honeybee.. J Neurosci.

[64] Farooqui T, Vaessin H, Smith BH (2004). Octopamine receptors in the honeybee (*Apis mellifera*) brain and their distribution by RNA-mediated interference.. J Insect Physiol.

[65] Takahashi T, Hamada A, Miyawaki K, Matsumoto Y, Mito T (2009). Systemic RNA interference for the study of learning and memory in an insect.. J Neurosci Methods.

[66] Tzschentke TM (2007). Measuring reward with the conditioned place preference (CPP) paradigm: update of the last decade.. Addiction Biol.

[67] Srinivasan MV (2010). Honey bees as a model for vision, perception, and cognition.. Ann Rev Entom.

[68] Niggebrügge C, Leboulle G, Menzel R, Komischke B, Hempel de Ibarra N (2009). Fast learning but coarse discrimination of colours in restrained honey bees.. J Exp Biol.

[69] Sokal RR, Rohlf RJ (1995). Biometry: the principles and practice of statistics in biological research. 3rd edition.

[70] Abramson CI, Giray T, Mixson TA, Nolf SL, Wells H (2010). Proboscis conditioning experiments with honeybees, *Apis mellifera caucasica*, with butyric acid and DEET mixture as conditioned and unconditioned stimuli.. J Insect Sci.

[71] Hawes JJ, Brunzell DH, Narasimhaiah R, Langel U, Wynick D (2008). Galanin protects against behavioral and neurochemical correlates of opiate reward.. Neuropsycopharmacol.

[72] Giray T, Giovanetti M, West-Eberhard MJ (2005). Juvenile hormone, reproduction, and worker behavior in the neotropical social wasp *Polistes canadensis*.. Proc Natl Acad Sci U S A.

