# Supplementary figures and images for: Dopamine and Octopamine Influence Avoidance Learning of Honey Bees in a Place Preference Assay
Source: PLoS One. 2011 Sep 30;6(9):e25371. doi: 10.1371/journal.pone.0025371 (PMC3184138; doi:10.1371/journal.pone.0025371)

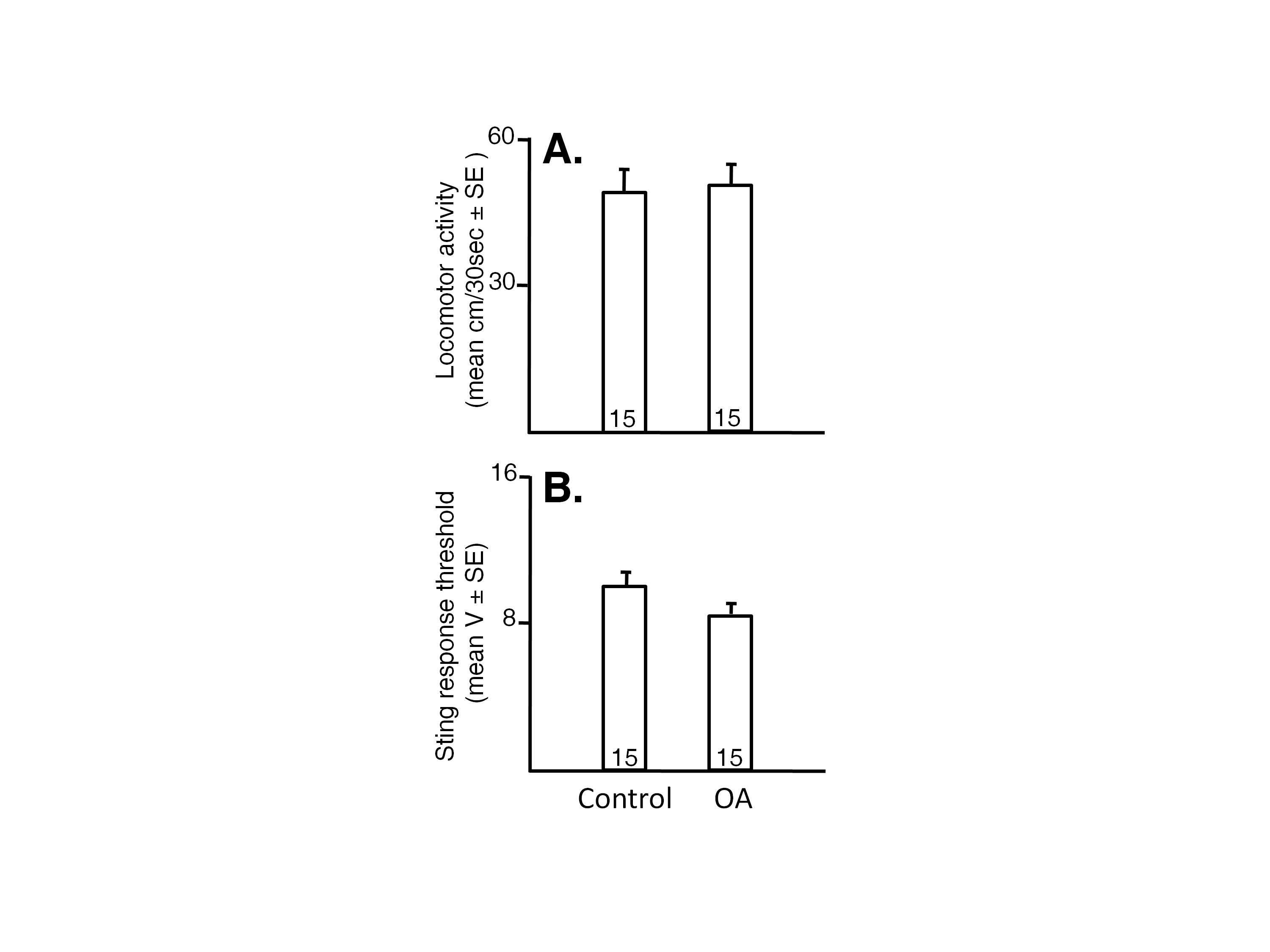

Supplement: Figure S1 — Locomotor activity and sting response threshold of bees. A. Locomotor activity of bees in the OA treatment and control groups were statistically not different (t-test: t = −0.286; df = 28; P>0.77), measured as distance walked (cm) in unit time (30 sec.) by each bee in each lane of the assay chamber in absence of electric shock. B. Sting response threshold of bees in the OA and control treatment groups were statistically not different (t-test on log transformed data: t = 1.691; df = 28; P>0.10), measured as the least amount of electric shock (V) that resulted in sting extension response for bees tested individually in the assay chamber. The numbers in the bars indicate the number of individuals in each group. (TIF) [file pone.0025371.s001.tif]

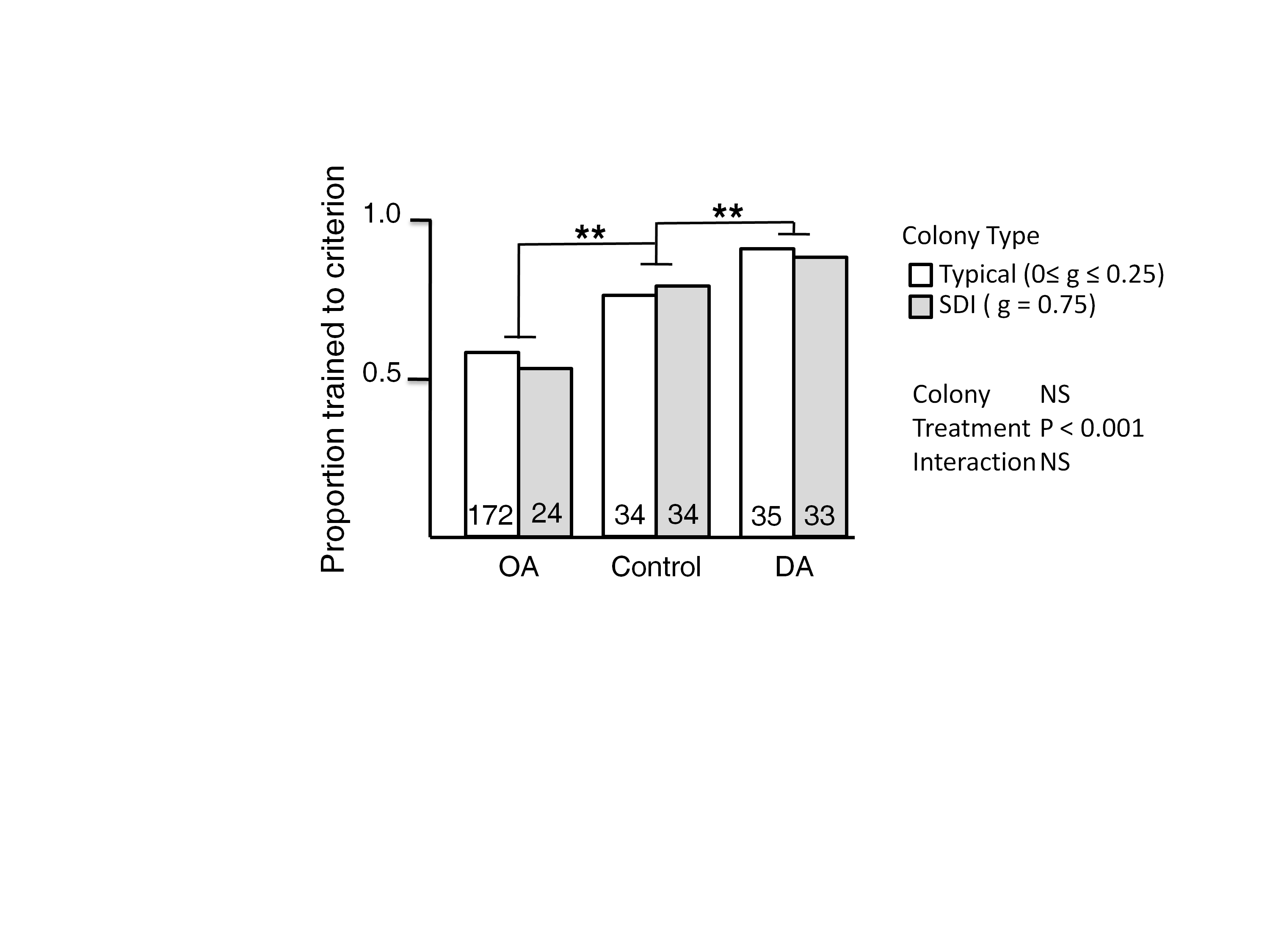

Supplement: Figure S2 — Genetic effects on variation in learning performance in the place preference assay. Experiments were performed with genetically similar bees obtained from a queen that was instrumentally inseminated by semen from a single drone ( SDI colony, genetic relatedness coefficient g = 0.75) to see if the variations in learning were due to higher genetic variation found in typical colonies (typical colony, across colonies, g = 0). Comparison of SDI colony or typical colony bees for proportion of individuals trained to criterion in principal treatment groups (control, OA, DA), demonstrate that results are similar for both types of bees (Wald test: Genetic similarity: X2 = 0.174, df = 1, P = 0.68; Treatment: X2 = 18.969, df = 2, P<0.0001; Genetic similarity and treatment interaction X2 = 0.469, df = 2, P = 0.79). The main effects of treatments with OA where lower proportion of bees were trained to complete avoidance, and DA where higher proportion of bees were trained to complete avoidance, were similar in direction and magnitude to the pooled data for bees from different colonies with naturally mated queens. (TIF) [file pone.0025371.s002.tif]
